# Increased Risk of Temporomandibular Joint Closed Lock: A Case-Control Study of ANKH Polymorphisms

**DOI:** 10.1371/journal.pone.0025503

**Published:** 2011-10-07

**Authors:** Boyen Huang, Katsu Takahashi, Tomoko Sakata, Honoka Kiso, Manabu Sugai, Kazuma Fujimura, Akira Shimizu, Shinji Kosugi, Tosiya Sato, Kazuhisa Bessho

**Affiliations:** 1 Department of Oral and Maxillofacial Surgery, Graduate School of Medicine, Kyoto University, Kyoto, Japan; 2 Translational Research Center, Kyoto University Hospital, Kyoto University, Kyoto, Japan; 3 Department of Biomedical Ethics, Graduate School of Medicine, Kyoto University, Kyoto, Japan; 4 Department of Biostatistics, School of Public Health, Kyoto University, Kyoto, Japan; University of Pittsburgh, United States of America

## Abstract

**Objectives:**

This study aimed to carry out a histological examination of the temporomandibular joint (TMJ) in *ank* mutant mice and to identify polymorphisms of the human ANKH gene in order to establish the relationship between the type of temporomandibular disorders (TMD) and ANKH polymorphisms.

**Materials and Methods:**

Specimens from the TMJ of *ank* mutant and wild-type mice were inspected with a haematoxylin and eosin staining method. A sample of 55 TMD patients were selected. Each was examined with standard clinical procedures and genotyping techniques.

**Results:**

The major histological finding in *ank* mutant mice was joint space narrowing. Within TMD patients, closed lock was more prevalent among ANKH-OR homozygotes (p = 0.011, OR = 7.7, 95% CI 1.6–36.5) and the elder (p = 0.005, OR = 2.4, 95% CI 1.3–4.3).

**Conclusions:**

Fibrous ankylosis was identified in the TMJ of *ank* mutant mice. In the human sample, ANKH-OR polymorphism was found to be a genetic marker associated with TMJ closed lock. Future investigations correlating genetic polymorphism to TMD are indicated.

## Introduction

Temporomandibular disorders (TMD) are a developing issue in public health with clinical signs being observed in 50% of the population [1], [2]. Analysis of data derived from clinical records has found that the majority of symptomatic TMD patients exhibited internal derangement of the temporomandibular joint (TMJ) [3]. Treatment outcomes of TMJ internal derangement remain controversial although various management options such as arthroscopy, arthrocentesis and physiotherapy have been suggested [4]–[6].

Internal derangement has been associated with 90% of cases suffering from TMJ closed lock [4]. Closed lock was identified as a permanently displaced disc and direct condyle articulation against a vascularised posterior disc attachment [7]. This manifestation generally emerged as a progression from TMJ clicking, pain and/or intermittent locking [4]. Although internal derangement was found to be preceded by TMJ clicking [4], TMJ clicking did not predispose closed lock [8]. TMJ clicking and closed lock showed contrasting tissue reactions [7]. The conditions also differed in their correlations to Type I TMJ ankylosis [9], a disorder characterised by articular fibrous adhesions in the TMJ [10]. In addition, compared to the cases with TMJ clicking, those that had progressed to closed lock were more likely accompanied with osteoarthritis [11]. These findings implied a pathological divergence between closed lock and TMJ clicking.

The over-expression of certain genes such as lumican [12], TRAIL and DR5 [13] has been detected in TMJ discs exhibiting internal derangement. A nonsynonymous mutation of the COMT gene was identified in a patient with TMJ closed lock [14]. In spite of these early findings, genetic influences on TMJ internal derangement require further investigation. In further research, a higher frequency of TMJ internal derangement and degenerative changes has been reported in patients with ankylosing spondylitis [15]. Recent studies have proposed *ank* mutations and ANKH polymorphisms, respectively, as determinants for arthritis in mice [16] and ankylosing spondylitis in humans [17]. The ANKH gene is a human homolog of the murine progressive ankylosis gene, *ank* [17]. Although the TMJ was susceptible to the above ANKH-related diseases [15], [18], [19], the connection between TMD and the ANKH gene remains unknown.

Since genetic effects on TMJ internal derangement have not been fully clarified and the progressive ankylosis gene was associated with joint disorders, an investigation of this gene may help to gain insights into the pathogenesis of TMD. Therefore, this study aimed (1) to carry out a histological examination of the TMJ collected from *ank* mutant as well as wild-type mice, and (2) to conduct a case-control research study to identify polymorphisms of the ANKH gene, using a sample of TMD patients in Japan. A special interest was to establish a relationship between the types of TMJ internal derangement and ANKH polymorphisms.

## Materials and Methods

Prior to commencement, animal and human sub-projects of this study have both received appropriate ethics approval from the Institutional Review Board of Kyoto University (approved ID Number: G86). Commercially bred *ank*/*ank* mutant and wild-type mice (Jackson Laboratory, Bar Harbor, ME, USA, http://jaxmice.jax.org/strain/000200.html) were purchased for the animal experiment. The mice were separately euthanised with carbon dioxide gas at 3, 4 and 5 months of age, since the life span of *ank*/*ank* mutant mice was less than 6 months [20]. Tissues of the TMJ were collected and prepared. The number of tissue sections (8 µm) per TMJ ranged from 50 to 150. Every 5th sequenced section was examined with a haematoxylin and eosin staining (HE stain) method [21].

To identify the expression of ANKH in TMJ, an mRNA expression analysis was conducted. Synovial cells used for this purpose were collected from a patient undergoing TMJ arthroscopy due to closed lock, with appropriate written informed consent received. Procedures for preparation of the primary culture and the reverse transcriptase polymerase chain reaction (RT-PCR), as suggested in literature [22], were used. This study applied AnkRTF4 (5′-ATCAAGAAGTTCACCTTCGTC-3′) and AnkRTR4 (5′-CTTTTTCTGCTTCCGGTAGAC-3′) as the primers to perform the RT-PCR technique.

This study hypothesised that closed lock patients were more likely to carry a homozygous ANKH polymorphic genotype. The calculation of a minimal sample size based on the statistical power to hypothesis testing was unattainable, since the homozygosity/heterozygosity distribution of the ANKH polymorphisms studied has never been reported. The percentage of controls carrying a homozygous ANKH polymorphism would be required for estimating the sample size of such an unmatched case-control study. To achieve a satisfactory sample size for an adequate statistical power, sample collection was carried out from January 2003 to December 2006. A sample was recruited for the study at the outpatient clinic of oral and maxillofacial surgery, Kyoto University Hospital. Inclusion criteria for subjects of the study were: (1) having a Japanese ethnicity background; (2) being older than 15 years of age; (3) presenting signs and symptoms that were characteristic of a diagnosis of TMJ clicking (disc displacement with reduction) or closed lock (disc displacement without reduction) [23], [24]; and (4) not having a previous history of trauma, neoplasm and/or surgery in the TMJ. The assessment consisted of a standardised clinical evaluation of mandibular range of motion, joint pain, joint sounds [23] and a magnetic resonance imaging (MRI) examination using a 1.5-T MRI scanner with bilateral 3-inch dual-surface coils (Signa, GE Healthcare, Little Chalfont, UK). Based on diagnoses, subjects were categorised as closed lock or TMJ clicking. Participants appearing with intermittent lock, a temporary and recurrent limitation of mouth opening [25], were excluded from the study, since proceeding developments of the indefinite manifestation ranged from a disc displacement with reduction to a complete disappearance of symptoms [25]. As absence of clicking sound failed to indicate a normal joint [25], [26] and this study focused on comparison between the two types of internal derangement, TMJ clicking patients instead of asymptomatic individuals were used as the controls. Closed lock subjects, on the other hand, were used as the cases. Before inclusion, written informed consent was received from all patients. Clinical and demographic data of each participant, including the type of TMJ internal derangement (closed lock or clicking), age (years) and gender (male or female), were collected. Blood samples of the patients were obtained and then prepared for DNA isolation. Total genomic DNA from peripheral leukocytes was extracted with a QIAamp DNA Blood Midi Kit (QIAGEN, Hilden, Germany).

The genotyping techniques reported by a previous study were used to identify the ANKH-OR polymorphic site and the ANKH-TR polymorphic site in the 5′-noncoding region and the promoter region of the ANKH gene, respectively [17]. The polymerase chain reaction (PCR) procedure for genotyping was carried out with application of LA Taq with GC Buffer (TaKaRa, Tokyo, Japan). This was initiated with 30 cycles of 30-second denaturation at 94°C, followed by 30-second annealing at 60°C and 2-minute extension at 72°C, in a GeneAmp PCR System 9700 thermal cycler (Applied Biosystems, Foster City, CA, USA). After sequencing the isolated PCR products with a BigDye Terminator v1.1 Cycle Sequencing Kit (Applied Biosystems, Foster City, CA, USA), the sequencing reaction products were purified with a Centri-Sep Spin Column (Princeton Separations, Freehold, NJ, USA). Sequences of the sense and the antisense strands in the above products were then obtained, using a 3100 Genetic Analyzer (Applied Biosystems, Foster City, CA, USA). Thus, polymorphisms of the ANKH gene in each sample were detected. Based on combinations of alleles, the variables of genotypes studied included ANKH-OR (homozygotes or heterozygotes) and ANKH-TR (homozygotes or heterozygotes) polymorphisms. All procedures were carried out according to the manufacturers' instructions.

Data entry and statistical analysis were implemented with JMP 8.0 (SAS Institute Inc., Cary, NC, USA, 2008). Data analysis included descriptive statistics (frequency distribution and cross tabulation). A univariate logistic regression method was used to assess the individual (unadjusted) contribution of explanatory variables including genotypes of ANKH polymorphisms, gender and/or age [27]. Variables that showed statistical significance in the univariate analysis were selected to enter a multivariate logistic regression model to examine the collective (adjusted) effect [27]. As highly correlated variables included in a model together might lead to overestimation or understimation of their significance [27], correlated ANKH-OR and ANKH-TR [17] were separatedly added into two different models. The level of two-sided significance was set at 5%.

## Results

**Figure 1:**
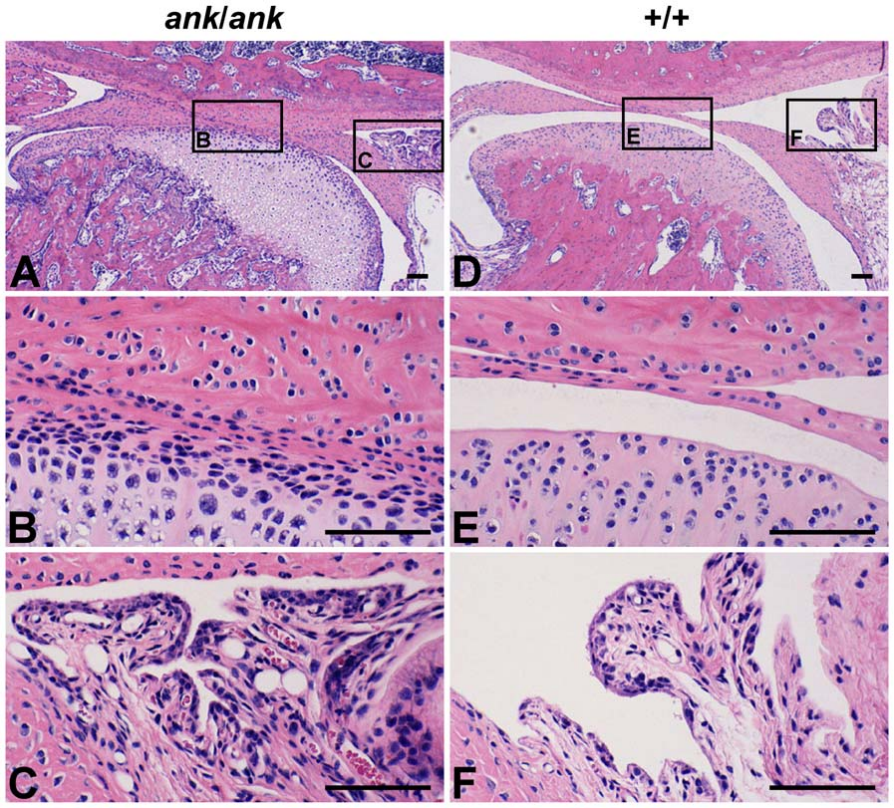
Representative HE stain photomicrographs of the TMJ from *ank*/*ank* mutant and wild-type mice. (A to C) Representative HE stain photomicrographs of the TMJ from a 3-month-old *ank*/*ank* mutant mouse of the study. (A) Narrower and/or ankylosed superior and inferior synovial cavities filled with fibrous connective tissue were visible. (B) A closer view of the ankylosed/adhered joint space. (C) Inflammatory cells appeared in the synovial membrane of one but not all mice. (D to F) Representative HE stain photomicrographs of the TMJ from a 3-month-old wild-type mouse of the study. (D) A normal and clear joint space was shown. (E) A closer view of the normal joint space. (F) The retrodiscal tissue was in a normal condition. Depth of tissue section in all photomicrographs: 3385 µm mesial to the most buccal point at the intersection of the zygomatic and the temporal bones. Scale bars in all photomicrographs: 100 µm.

**Figure 2:**
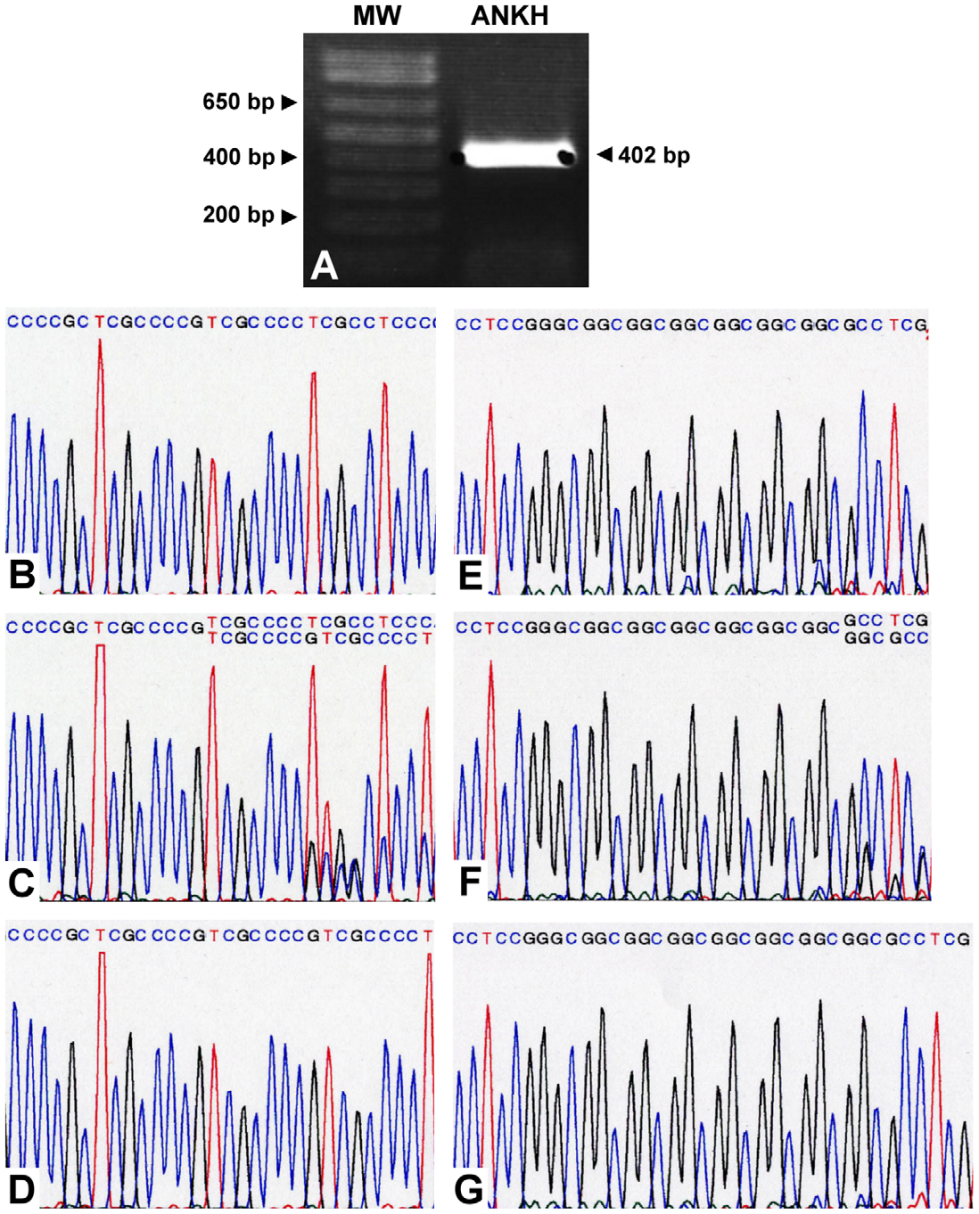
Results of ANKH mRNA expression in the TMJ and sequence traces of ANKH polymorphic sites. (A) Assessment of ANKH mRNA expression in RT-PCR. The sizes of the molecular weight markers (MW) are displayed on the left. Sizes are given in base pairs (bp). (B to G) Sequence traces of identified polymorphic sites on the ANKH gene in the human sample of the study. (B) Genotype 1/1 of ANKH-OR. (C) Genotype 1/2 of ANKH-OR. (D) Genotype 2/2 of ANKH-OR. (E) Genotype 7/7 of ANKH-TR. (F) Genotype 7/8 of ANKH-TR. (G) Genotype 8/8 of ANKH-TR.

In both the *ank*/*ank* mutant and the wild-type mice, six 3-month-olds, two 4-month-olds and two 5-month-olds were examined. Mutant individuals of all age groups showed similarly narrower and/or ankylosed superior and inferior synovial cavities filled with fibrous connective tissue throughout the entire joint space (Figure 1: A–C). This was not seen in any of the wild-type mice (Figure 1: D–F). Thicker fibrocartilage in the condylar head and inflammatory cell infiltration in the synovial membrane were identified in one (Figure 1: A–C) but not all *ank*/*ank* mutant mice. Osseous ankylosis, bone/cartilage erosion, calcified debris and joint calcification were not found in the TMJ of either genotype amongst any age groups. As for the human TMJ, the primary culture of cells derived from the surgically removed synovial membrane expressed ANKH at the mRNA level (Figure 2: A).

From January 2003 to December 2006, five-hundred-and-forty suspected cases of TMJ internal derangement attended the outpatient clinic of oral and maxillofacial surgery, Kyoto University Hospital. One-hundred-and-fifty-one patients completed both clinical and MRI examination sessions. Excluding 17 cases with an inconsistent diagnosis, internal derangement was confirmed by both assessments in a total of 134 patients. These included 61 cases with locking and 73 cases without locking but clicking. All of the 134 patients were from a Japanese ethinicity background, being older than 15 years of age, and having no previous history of trauma, neoplasm and/or surgery in the TMJ. Fifty-two locking cases and 16 clicking cases consented to participate, providing a response rate of 85.2% and 21.9%, respectively. These contributed to a general response rate of 50.7%. Thirteen locking patients were excluded from data analysis due to their appearing with intermittent lock of the TMJ. The final sample included 55 cases. Of these patients, forty-four (80.0%, 95% CI: 69.4%–90.6%) were female. The participants' age ranged from 15.5 to 69.7 years and the mean age of female and male subjects was 39.7±18.1 years and 30.8±12.1 years, individually. Thirty-nine (70.9%, 95% CI: 58.9%–82.9%) and 16 (29.1%, 95% CI: 17.1%, 41.1%) cases were separately diagnosed with closed lock and TMJ clicking (Table 1).

**Table 1:** Frequency distribution of TMJ internal derangement by gender, age and genotypes of ANKH polymorphisms in the sample of the study (n = 55).

	Closed lock [n (%)]	Clicking [n (%)]	All [n (%)]	Unadjusted OR^a^ (95% CI)	p-values	Adjusted OR^b^ (95% CI)	p-values	Adjusted OR^c^ (95% CI)	p-values
Genotypes of ANKH-OR polymorphisms									
Heterozygotes	13 (52.0%)	12 (48.0%)	25 (45.5%)	1				1	
Homozygotes	26 (86.7%)	4 (13.3%)	30 (54.5%)	6 (1.6–22.3)	0.005			7.7 (1.6–36.5)	0.011
Genotypes of ANKH-TR polymorphisms									
Heterozygotes	17 (60.7%)	11 (39.3%)	28 (50.9%)	1		1			
Homozygotes	22 (81.5%)	5 (18.5%)	27 (49.1%)	2.9 (0.8–9.8)	0.090	1.9 (0.5–7.9)	0.363		
Gender									
Female	34 (77.3%)	10 (22.7%)	44 (80.0%)	1		1		1	
Male	5 (45.5%)	6 (54.5%)	11 (20.0%)	0.3 (0.1–0.96)	0.038	0.3 (0.1–1.4)	0.130	0.3 (0.1–1.7)	0.174
Age									
	43.0±17.2^d^	25.6±10.6^d^	38.0±17.4^d^	2.2 (1.3–3.7)^e^	0.003	2.1 (1.2–3.6)^e^	0.009	2.4 (1.3–4.3)^e^	0.005

a results based on univariate logistic regression statistics.

b results based on multivariate logistic regression statistics when excluding genotypes of ANKH-OR polymorphisms.

c results based on multivariate logistic regression statistics when excluding genotypes of ANKH-TR polymorphisms.

d mean ± standard deviation (years of age).

e each additional 10 years of age.

Two alleles of the ANKH-OR polymorphism were identified, including TCGCCCCG (Allele-1) and TCGCCCCGTCGCCCCG (Allele-2) located in the putative 5′-noncoding region (Figure 2: B–D). According to the combinations of the alleles, there were 7 (12.7%) Allele-1 homozygotes (Genotype 1/1), 23 (41.8%) Allele-2 homozygotes (Genotype 2/2) and 25 (45.5%) Allele-1/Allele-2 heterozygotes (Genotype 1/2) in this sample. Two alleles of the ANKH-TR novel polymorphism were detected in the promoter region, including 7 (Allele-7) and 8 (Allele-8) copies of GGC repeats, respectively (Figure 2: E–G). Combinations of these alleles contributed to 8 (14.5%) Allele-7 homozygotes (Genotype 7/7), 19 (34.6%) Allele-8 homozygotes (Genotype 8/8) and 28 (50.9%) Allele-7/Allele-8 heterozygotes (Genotype 7/8). According to the above, thirty (54.5%, 95% CI: 41.4%–67.7%) ANKH-OR and 27 (49.1%, 95% CI: 35.9%–62.3%) ANKH-TR homozygotes were identified.

Statistically significant variables in the univariate analysis included ANKH-OR (p = 0.005, OR = 6.0, 95% CI 1.6–22.3), ANKH-TR (p = 0.090, OR = 2.9, 95% CI 0.8–9.8), gender (p = 0.038, OR = 0.3, 95% CI 0.1–0.96) and age (p = 0.003, OR = 2.2, 95% CI 1.3–3.7). In the multivariate regression model, participants that were ANKH-OR homozygotes (Genotype 1/1 or Genotype 2/2) showed a higher risk of TMJ closed lock than those who were ANKH-OR heterozygotes (Genotype 1/2) (p = 0.011, OR = 7.7, 95% CI 1.6–36.5) (Table 1). With the sample size used and a two-sided significance level at 5%, the explanatory power of the test was 0.83, which indicated an adequate statistical power [28]. Older patients more likely sustained closed lock (p = 0.005, OR = 2.4, 95% CI 1.3–4.3) (Table 1). Gender and genotypes of ANKH-TR polymorphisms were not related to the type of TMJ internal derangement (p≥0.130) (Table 1).

**Figure 3:**
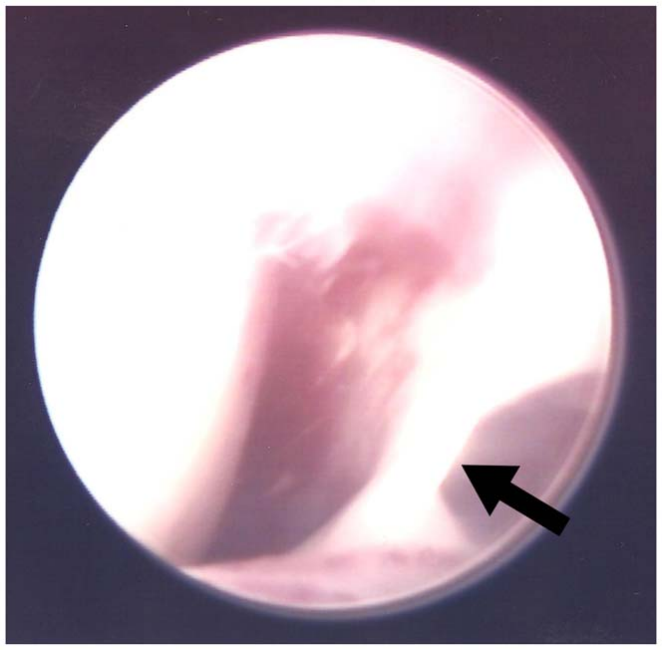
Pre-operative arthroscopic view of the right TMJ from an ANKH-OR homozygote (Genotype 2/2) with closed lock. The white strip at the right portion of the view indicated fibrous tissues adhering the joint (arrow).

Figure 3 shows the right TMJ of an ANKH-OR homozygous (Genotype 2/2) participant with closed lock. Type I TMJ ankylosis (fibrous adhesion) was identified in the pre-operative arthroscopic view. Quntitative analysis of the arthroscopic data was unattainable as only 2 patients with closed lock in this sample consented to have their arthroscopic images used for research and demonstration. None of the TMJ clicking subjects were managed with this invasive procedure.

## Discussion

Fibrous ankylosis was notably found in the TMJ of *ank*/*ank* mutant mice. Joint space narrowing found in this group represents a relevant pathological trait of fibrous ankylosis [29] and/or osteoarthritis [30]. This finding was consistent with a previous study reporting narrowed joint spaces in the hind limb interphalangeal joints of *ank*/*ank* mutant mice [16]. Past studies have identified the ANKH protein as serving a critical role in the transport of pyrophosphate ions (PPi) [31] which inhibit ectopic mineralisation in bones [32] as well as joints [33]. Although it was not found in this study, an impaired *ank* function has been associated with calcified debris, excessive calcification and cartilage erosion in the joints of *ank*/*ank* mutant mice [16]. The discrepancy in the occurrence of calcified changes could be due to a concurrence of crystal deposition and osteoarthritis in most synovial joints except the TMJ [34]. Utilisation of 3-to-5-month-old mice in this study might be responsible for the absence of erosive changes in the TMJ. According to a biglycan and fibromodulin double-deficient mouse model, osteoarthritis-related cartilage and bony defects in the TMJ were not identified until the age of 9 months although some histological changes in the condylar cartilage were found amongst 3-month-olds [35]. With the exception of joint space narrowing, histological signs of osteoarthritis [36] and synovitis [37], such as joint calcification, joint erosion, proliferative fibrocartilage and lymphocyte infiltration, were rarely seen in this study. Thus, this study could not direct its findings to an association with osteoarthritis and/or synovitis. Consequently, a connection can only be inferred between mutations of the murine *ank* gene and fibrous ankylosis of the TMJ.

This study has demonstrated for the first time an enhancing effect of homozygous ANKH-OR polymorphisms on closed lock of the human TMJ. ANKH-OR homozygotes were approximately 8 times more likely to develop closed lock than their control group counterparts. A past study has reported a more serious phenotype of craniometaphyseal dysplasia amongst homozygous *ank* mutant mice than their heterozygous littermates, based on an autosomal dominant trait of the disorder [38]. Since both types of TMJ internal derangement were observed in all ANKH-OR genotypes, a higher morbidity of closed lock in the homozygous genotypes could be attributable to more complicated molecular mechanisms and/or genetic interactions. A previous study has suggested ANKH-OR and ANKH-TR polymorphisms to be in complete linkage disequilibrium [17]. The disproportionate numbers of ANKH-OR and ANKH-TR homozygotes in the sample of this current study did not corroborate complete linkage disequilibrium of these loci, although it should be noted that genetic interactions were not examined. The discrepancy encourages further investigation into expressions and interactions of ANKH polymorphisms.

Age was related to the type of TMJ internal derangement in this sample and this agreed with a previous study [39]. It was observed that closed lock occurred several months, or at times more than 10 years after milder TMJ symptoms [4]. This long incubation time could explain why closed lock was seen more frequently in older patients. Hence, the risk of a transit from TMJ clicking to closed lock could increase with age amongst those younger ANKH-OR homozygotes who did not exhibit the severer condition. However, a lower response rate could have resulted in a potential of sampling bias in this study. Application of a cohort study method would be able to explore the influence of age on TMD. Compared to closed lock, TMJ clicking was not gravely regarded. A high percentage of patients having joint sounds hesitated to participate in this study as they were unwilling to take a blood test to assess such an unproblematic condition. Buccal smear for DNA collection may be a more encouraging alternative. Since there are no previous studies using a buccal smear technique to examine ANKH polymorphisms, its feasibility requires further investigation.

This study identified ANKH expression in TMJ synovial cells. It was found that mutations and polymorphisms of the ANKH gene predisposed closed lock in humans and fibrous ankylosis in mice. Although this study did not indicate an equivalence between fibrous ankylosis in mice and closed lock in humans, past studies have reported relationships and similarities between the two disorders.. Fibrous adhesion, known as Type I TMJ ankylosis [10], has been noticed in a number of closed lock cases [4]. This fibrous change has shown a positive correlation with closed lock and a negative correlation with clicking [9]. The pathological duration of closed lock was related to the formation of fibrous adhesions, however TMJ clicking did not exhibit this relationship [40]. Of further note, inflammatory cells that were absent in most ankylosed murine TMJs in this study were also rarely seen in the human TMJs with internal derangement [41]. The ANKH gene, a suggested genetic marker for closed lock in this study, is a human homolog of the murine *ank* gene [17] which contributed to fibrous ankylosis in mice. Thus, it could be deduced that a mouse model is adequate for the simulation of human ANKH-related TMJ internal derangement. In humans, the ANKH gene has been associated with a variety of skeletal and joint defects including ankylosing spondylitis [17], cuff tear arthropathy [42], craniometaphyseal dysplasia [43] and calcium pyrophosphate dihydrate disease [44]. The ANKH protein has been found to be pertinent to the transport of PPi across the plasma membrane and it has been observed as a rescue to certain phenotypes of the above diseases [31]. Hence, future biochemical approaches to the role of PPi on TMD development and rescue are indicated.

### Conclusions

This study has manifested that the ANKH-OR polymorphism is a genetic marker associated with TMJ closed lock. ANKH expression in the TMJ has been confirmed. In addition, fibrous ankylosis in the TMJ of *ank* mutant mice has been identified by this study.

TMD has become an important issue in public health and dentistry. A genetic approach to the pathogenesis adds to current understanding of aetiology of TMD. Succeeding investigations into the influence of the ANKH gene on mechanisms of TMD development are indicated.
